# Overlapping gene dependencies for PARP inhibitors and carboplatin response identified by functional CRISPR-*Cas9* screening in ovarian cancer

**DOI:** 10.1038/s41419-022-05347-x

**Published:** 2022-10-28

**Authors:** Ricardo Coelho, Alessandra Tozzi, Muriel Disler, Flavio Lombardo, André Fedier, Mónica Núñez López, Florian Freuler, Francis Jacob, Viola Heinzelmann-Schwarz

**Affiliations:** 1 Ovarian Cancer Research, Department of Biomedicine, University Hospital Basel and University of Basel, Basel, Switzerland; 2 Hospital for Women, University Hospital Basel, Basel, Switzerland

**Keywords:** Ovarian cancer, DNA damage and repair

## Abstract

PARP inhibitors (PARPi) have revolutionized the therapeutic landscape of epithelial ovarian cancer (EOC) treatment with outstanding benefits in regard to progression-free survival, especially in patients either carrying *BRCA1/2* mutations or harboring defects in the homologous recombination repair system. Yet, it remains uncertain which PARPi to apply and how to predict responders when platinum sensitivity is unknown. To shed light on the predictive power of genes previously suggested to be associated with PARPi response, we systematically reviewed the literature and identified 79 publications investigating a total of 93 genes. The top candidate genes were further tested using a comprehensive CRISPR-*Cas9* mutagenesis screening in combination with olaparib treatment. Therefore, we generated six constitutive *Cas9*^+^ EOC cell lines and profiled 33 genes in a CRISPR-*Cas9* cell competition assay using non-essential (*AAVS1*) and essential (*RPA3* and *PCNA*) genes for cell fitness as negative and positive controls, respectively. We identified only *ATM, MUS81, NBN, BRCA2*, and *RAD51B* as predictive markers for olaparib response. As the major survival benefit of PARPi treatment was reported in platinum-sensitive tumors, we next assessed nine top candidate genes in combination with three PARPi and carboplatin. Interestingly, we observed similar dropout rates in a gene and compound independent manner, supporting the strong correlation of cancer cell response to compounds that rely on DNA repair for their effectiveness. In addition, we report on *CDK12* as a common vulnerability for EOC cell survival and proliferation without altering the olaparib response, highlighting its potential as a therapeutic target in EOC.

## Introduction

The approval of the poly (ADP-ribose) polymerase inhibitor (PARPi) olaparib in 2014 as a treatment option in patients with germline or somatic *BRCA1/2*-mutation (g/s*BRCA 1/2*) and more than three prior lines of chemotherapy [1] has transformed the treatment landscape of EOC, particularly in the maintenance setting. PARPi are designed to compete with NAD^+^ for the catalytically active site of PARP molecules leading to the persistence of unrepaired DNA single-strand breaks by trapping PARP molecules and consequently resulting in DNA double-strand breaks by the collapse of stalled replication forks [2, 3]. Double-strand DNA breaks are repaired by either error-free homologous recombination (HR) or error-prone non-homologous end-joining (NHEJ) [4, 5]. Thus, the insufficient repair of DNA double-strand breaks in HR-deficient (HRD) cancer cells displays an optimal target for PARPi and consequently results in chromosome alterations and ultimately cell death [6, 7]. This vulnerability has been successfully applied in HR-deficient tumors treated with PARPi, resulting in synthetic lethality. This mechanism also accounts for the enhanced benefit in patients with *BRCA1/2* mutations [8, 9].

Several approaches have been suggested to detect HR-deficiency (HRD), including scores to measure the effects on genome stability, namely loss-of-heterozygosity, large-scale transitions, and telomeric allelic imbalance, collectively referred to as “genomics scars” [10–12]. Still, the presence of genomic scars only reflects a snapshot of HR, failing to evaluate the dynamic changes that may occur during tumor formation and evolution. In fact, reversion mutations restoring the HR functionality in genes such as *BRCA1/2* [13–15], *RAD51C*, and *RAD51D* [16] have been reported in several cancers and are associated with resistance to PARPi and chemotherapy. Although *BRCA* mutations and HR deficiency tests represent important predictive markers for PARPi response, the extended benefit of PARPi in EOC patients was also reported in *BRCA* wild-type and HR-proficient tumors in several clinical trials [17–19], highlighting the complex molecular mechanisms underlying PARPi efficacy.

HR deficiency is also associated with sensitivity to other DNA-damaging agents [20, 21], and platinum sensitivity in EOC is a strong clinical predictor for the benefit of PARPi treatment [22, 23], suggesting a common mechanism of response. Three PARPi are now licensed for the treatment of EOC, however, there is still a persistent debate in deciding which PARPi to choose in which line of treatment. The clinical application of the different PARPi is mainly based on the outcome of clinical trials and the adverse drug reactions of the different inhibitors’ potential arising from their differential polypharmacology [24, 25]. Therefore, it is of utmost importance to perform a comprehensive investigation on the potential differential gene dependencies for response to the different PARPi and platinum chemotherapy.

For this purpose, our study comprehensively assessed the current literature reporting on genes functionally linked to PARPi response. Identified and curated gene candidates were systematically examined on their role in cancer cell fitness and olaparib response upon genetic deletion using a functional CRISPR-*Cas9* mutagenesis assay with further validation in other PARPi and carboplatin chemotherapy.

## Materials and methods

### Literature search and quantitative evaluation

The PubMed database (https://www.ncbi.nlm.nih.gov/) was assessed to identify publications on ovarian cancer and PARP inhibitors during a 16-year period, from 2005 to July 2021. Thus, the literature search utilized the following terminologies: “ovarian cancer (cells)” and “PARP inhibitor”, “PARP inhibition”, “PARP”, “Olaparib”, “Niraparib”, “Talazoparib”, “Veliparib”, “Rucaparib”, “PARP inhibitors molecular mechanisms of action”, “gene mutation sensitivity”, “PARP inhibitors target in *BRCA* proficient cells”, “markers of response to PARP inhibition”, “PARP inhibitors in non-HR deficient tumor”, “off target PARP inhibitors toxicity”, “PARP inhibitors gene mutation”; AND: “gene silencing”, “knockout”, “knock out”, “deletion”; OR: “on target”, “off target”, “targeted therapies”, “targeted therapy”, “CRISPR”; “cancer genomic”, “cancer genomics”. Since our search aimed to identify genes that may be related to PARPi response, we decided to broaden our inclusion criteria to gynecologic carcinomas. Selected publications include human-derived cell lines or human clinical cancer samples of gynecological carcinomas including breast, cervical, endometrial cancer, and ovarian cancer.

### EOC cell lines, culture conditions, and compounds

EOC cell lines OVCAR3, OVCAR5, OVCAR8, ES-2, TYK-nu, and IGROV1 were purchased from ATCC, JCRB cell bank, and Oncotest GmbH (now Charles River Laboratories Inc, USA). All cancer cell lines were cultured in RPMI-1640 growth medium supplemented with 10% FBS (Sigma-Aldrich/Merck, Switzerland), 100 U/mL penicillin, and 0.1 mg/mL streptomycin (all purchased from Sigma-Aldrich/Merck). Cell lines were STR-profiled (Microsynth AG, Switzerland) and regularly tested for the absence of *Mycoplasma*. PARPi and carboplatin were obtained from various supplies: Sigma-Aldrich/Merck (olaparib, niraparib, and carboplatin), Selleckchem (talazoparib). Compounds were dissolved in DMSO (olaparib, niraparib, and talazoparib), or water carboplatin and stored as aliquots at −20.

### Publicly available data and computational analysis

IC_50_ data relative to cell lines were obtained from the depmap portal https://depmap.org/portal/download/custom/ (accessed in January 2022). These data were filtered for the following compounds: olaparib, niraparib, talazoparib, and cisplatin to generate Fig. 4C. Microsatellite instability data and cell line mutational data were obtained from depmap portal [26]. Homologous recombination status for the different cell lines was obtained from different publications [27–35]. Copy number and gene alterations were obtained from cBioPortal for the following genes: *53BP1, AAVS1, PDS5B, ARID1A, ATM, ATR, AURKB, BARD1, BCL2L1, BCL2L2, BRCA1, BRCA2, BRD4, BRD9, CBLC, CCND1, CCNE1, CDCA8, CDK12, CDK4, CDK5, CDK9, CHEK1, CHEK2, DCLRE1C, DDB1, DNPH1, SEM1, DYNLL1, E2F7, EHMT1, EHMT2, FBXO5, ERCC3, ERCC8, EZH2, FEN1, FOXM1, GPBP1, HMGA2, HMGB2, HSP90AA1, INPP4B, XRCC5, LIG1, MAPK12, MET, MIR-107/107, MIR-493/5* *P, MIR-506/506, MIR-509-3/3* *P, MIR-622/622, MIR-96/96, MLH1, MLH3, MRE11, MUS81, MYC, NBN, NF1, GABPA, NRP1, PALB2, PARG, PARP1, PARP2, PARP3, PIK3CA, PCNA, PLK3, PNKP, POLQ, PTEN, RAD50, RAD51, RAD51B, RAD51C, RAD51D, RAD52, ATRX, RBBP8, REV1, RNASEH2A, RNASEH2B, RNF168, RPA1, RPA3, RPRD1B, SEM1, SHLD1, SHLD2, SRC, SSRP1, TSSK3, STK36, TIGAR, TOPBP1, TP53, TRIP12, TWIST1, USP13, USP15, XAB2, XPC, XRCC1, XRCC2, XRCC3, XRCC5, XRCC6*; data was then combined using cell line id.

### Molecular cloning

Single guide RNAs (sgRNA) targeting protein-coding genomic DNA sequences of target genes were designed using Benchling (Biology Software, 2021, retrieved from https://benchling.com). SgRNAs with high-quality scores were selected for editing of the target gene (Supplementary Table 1). Single strand oligonucleotides were purchased from Sigma-Aldrich and cloned into either LRG2.1 (addgene, #108098) or LRG2.1_mOrange (Addgene #124772) using the *BsmBI* endonuclease restriction site. Annealed oligonucleotides were ligated into the desired plasmid using the T4-DNA ligase (Promega, Switzerland) for subsequent expression of the sgRNA together with either EGFP or mOrange fluorescent proteins. Ligations were transformed into *Stbl3 E.coli* following ampicillin selection, ZR Plasmid Miniprep—Classic plasmid purification (ZYMO Research, Lucerna-Chem AG, Switzerland), and Sanger DNA sequencing (Microsynth, Switzerland) to confirm insertion of respective sgRNA using the human U6 primer (5′- GAG GGC CTA TTT CCC ATG ATT-3′).

### Generation of constitutive *Cas9*^+^ expressing ovarian cancer cell lines and delivery of single-guide RNAs

Human Embryonic Kidney HEK293T cells were seeded in a T75 flask at 50% confluency one day before transfection. A total of 4 μg of LRG2.1 (Addgene #108098) or LRG2.1_mOrange (Addgene #124772) encoding the sgRNA of interest or LentV-Cas9-puro (Addgene #108100), 2 μg of pMD2.G (Addgene #12259), and 2 μg of pCMVR8.74 (Addgene #22036) were co-transfected using 24 μL of jetPEI reagent in 1 mL of 150 mM NaCl solution (Polyplus-transfection, Chemie Brunschwig AG, Switzerland). The growth medium was changed 24 h after transfection. Supernatant containing lentivirus particles was collected 48 h later and filtered with a 0.45 μm polyvinylidene fluoride filter (Sartorius AG, Germany), aliquoted in 1.5 ml cryotubes, and stored at −80 °C until further use. Transduced cells were selected with 1–3 µg/mL puromycin for 1 week. Selected *Cas9*^+^ cell lines were kept in media containing 1 µg/ml puromycin except for downstream competition assays.

### Flow cytometry-based competition assay

Stable *Cas9* expressing cells were transduced with lentivirus containing sgRNAs targeting selected genes (Supplementary Table 1). SgRNAs targeting the same gene were used either individually or pooled and further diluted in a 1:5 or 1:8 ratio with a complete culture growth medium. The medium was changed 24 h after transduction and cells were incubated for 2 additional days until measuring the EGFP^+^ or mOrange^+^ cells. The percentage of fluorescence-positive cells was determined initially (baseline reference, passage 0) and all following passages using the CytoFLEX Flow Cytometer (Beckman Coulter, USA). In each passage, cells were washed with FACS-wash (FW-1% FBS in PBS) in 96-well V-shape plates and stained with DAPI (0.1 µg/ml) (Sigma-Aldrich/Merck, Switzerland) for 5 min. All investigated cell lines were gated individually to exclude debris, doublets, and dead (DAPI^+^) cells.

Delivery of drugs at passage 1 was performed with different PARPi (olaparib, niraparib and talazoparib) and carboplatin using a concentration causing approximately 10% growth inhibition (IC_10_) [36] for additional five passages. Dropout values represent the fold-change of the percentage of EGFP^+^ or mOrange^+^ cells at each passage, relative to passage 0 (3 days after transduction) and was used as a readout of effects on cell fitness or drug sensitivity conferred by the CRISPR-*Cas9* mediated gene mutations. Data analysis was performed using FlowJo v10 BD (Becton Dickinson, USA) and R/Bioconductor scripts. Of note, due to the high number of guides applied in this study and the number of cell lines investigated, each assay was performed at least in two independent experiments with the negative control *AAVS1* (non-essential gene) and at least one positive control (*RPA3* or *PCNA*). The time for cell passage up to 24 days was defined as the minimal time to observe an increase in the dropout higher than 1.7, for essential genes across the different cell lines (Supplementary Fig. 1A–E).

### Immunoblotting

EOC cell lines were lysed in 1x radioimmunoprecipitation assay buffer (RIPA, Cell Signaling Technology, BioConcept, Switzerland) containing proteinase inhibitor cocktails (Sigma-Aldrich/Merck, Switzerland). Lysates were clarified by centrifugation at 18,000 × *g* for 15 min at 4 °C. Clarified lysates were boiled in 1x sample buffer (50 mM Tris-HCl, 1% SDS, 100 mM DTT, and 10% glycerol) at 95 °C for 5 min and resolved by SDS-PAGE. Proteins were then transferred to a polyvinylidene difluoride (PVDF) membrane (BioRad, Switzerland) and blocked with 5% (w/v) bovine serum albumin in TBS-T (20 mM Tris-Base, 150 mM NaCl, pH 7.8, 0.1% Tween 20) for 1 h at room temperature. The membrane was incubated with one of the listed primary antibodies (Supplementary Table 2) diluted in 5% (w/v) BSA in TBS-T overnight at 4 °C. After washing (3 times, 10 min) in TBS-T, membranes were incubated with corresponding HRP-conjugated secondary antibodies (1:10,000, Cell Signaling, BioConcept, Switzerland) for 3 h at room temperature. Finally, the membrane was incubated with Super Signal West Dura Extended Duration Substrate (Thermo Fisher Scientific, Switzerland) for the detection of HRP. Western blot results were visualized by Gel Doc XR + TM (BioRad, Switzerland) and analyzed by Image Lab^TM^ software (BioRad, Switzerland). Original membranes can be found in Supplementary Fig. 2.

### Immunofluorescence

Cells were grown on eight-well tissue culture chamber slides (Sarstedt, Switzerland) for up to 48 h, fixed with 4% paraformaldehyde, and rinsed three times in PBS for 5 min each. For the evaluation of DNA repair capacity in *BRCA1*-edited cells, DNA damage was induced with 10 Gy y-radiation by a Caesium-137 source using the Gammacell 40 exactor (Best, Theratronics, Canada) and incubated for an additional 24 h followed by fixation with 4% paraformaldehyde. After fixation, cells were permeabilized with Triton X-100 0.25% in PBS for 5 min followed by incubation with blocking solution (5% FBS with 1% BSA in PBS 1% Triton-X 100) for 1 h. After blocking, cells were incubated with one of the listed primary antibodies (Supplementary Table 2) diluted in 1% BSA 1% Triton-X 100 in PBS and incubated overnight at 4 °C. Primary antibodies were detected using anti-rabbit IgG (H + L) Alexa Fluor 488, anti-rabbit IgG (H + L), anti-rabbit Alexa Fluor 555 Cell Signaling, anti-rabbit Alexa Fluor 647 Cell Signaling, or goat anti-mouse IgG (H + L) (Life Technologies, Thermo Fisher Scientific, Switzerland), secondary antibody diluted 1:500 in 1% BSA 1% Triton-X 100 in PBS for 3 h at room temperature. Slides were mounted using ProLong® Gold antifade reagent (Cell Signaling Technology, BioConcept Switzerland) and a coverslip. Images were taken using the confocal microscope Nikon CSU-W1, analyzed with Image J (2.3.0/1.53q) and Qupath (0.3.0) software, and developed scripts for cell detection and annotations. Further analyses were performed by R/Bioconductor.

### Analysis of *Cas9* activity using TIDE assay

Genomic DNA of *Cas9* expressing non-transduced (control) or transduced cells with sgRNAs targeting *AAVS1* (mock) sg1_AAVS1_5′-ACT GTT GAC GGC GGC GAT GT-3′, sg2_AAVS1_5′-GCT GAT ACC GTC GGC GTT GG-3′; *TP53* sg1_TP53_5′-AGA TGG CCA TGG CGC GGA CG-3; or RPA3 sg1_RPA3 5′-CCC AGG TCG CGC ATC AAC GC-3′ was extracted using the DNeasy Blood & Tissue Kit (Qiagen, Switzerland) 5 and 8 days after transduction. *Locus* targeted by *TP53, RPA3* guide RNAs were amplified using the primers listed in Supplementary Table 2 and 2x GoTaq (Promega, Switzerland). PCRs were performed using 2xGoTaq Green Master Mix (Promega, Switzerland), 200 nM of each primer, and 100 ng of genomic DNA. PCR conditions were initial DNA denaturation at 94 °C for 5 min followed by 32 cycles of 95 °C for 20 s, 62 °C for 15 s and 72 °C for 45 s with a final extension at 72 °C for 5 min. Amplicons were visualized on 1% agarose gel and purified by Wizard SV gel and PCR Clean/up System (Promega, Switzerland). Sequence traces were analyzed using the Tracking of Indels by Decomposition (TIDE) assay TIDE [37].

### Drug sensitivity and cell proliferation rate

Drug sensitivity was determined by the MTT and the colony formation assays. OVCAR3 and OVCAR8 *Cas9* expressing cells were transduced with lentivirus supernatant encoding sgRNAs targeting *AAVS1*, *ATM*, *BRCA1*, and *CDK12*. The percentage of cells harboring sgRNAs targeting the genes of interest was evaluated 3 days after lentivirus transduction and can be found in Supplementary Fig. 3. For the MTT-assay, 3000 cells in 200 µl of medium were seeded into 96-well plates and treated with each drug for 72 h: olaparib (range: 6.25-100 μM), and niraparib (range 1.56-50 µM). Then MTT-dye (cat. no. M2128; Sigma-Aldrich; final concentration: 0.5 mg/ml) was added for 3 h, followed by the removal of the medium and dissolution of the purple formazan crystals with DMSO. The optical density (OD; absorbance at 540 nm) was measured using the SynergyH1 Hybrid Reader (BioTek Instruments, Inc.). Data (mean ± SD) of at least two independent experiments from two independent lentiviral transductions are presented as the relative proliferation as a function of time after seeding. For the colony formation assay, 600 cells/ml culture medium were seeded into 12-well plates and exposed to olaparib on the next day for 8–10 days: 0.25, 0.5, 1 μM olaparib for OVCAR8-Cas9^+^ cells. Complete growth medium with and without drugs was replaced every 3 days. Then the medium was removed, and the colonies were fixed at room temperature for 1 h and stained with 0.05% crystal violet (Sigma-Aldrich) in 4% formalin (Formafix AG), the plates were then rinsed 3–4 times with water, dried and images of the plates were taken using the Fusion FX7 Edge Imaging System (Witek AG).

To evaluate proliferation, cells were seeded at 1500 cells/well density into 96 well plates and incubated for up to 4 days. At each time point, MTT dye was added at a final concentration of  0.5 mg/ml and incubated for 3 h and optical density was measured as mentioned above.

### Statistical analysis

Statistical analysis and figures were obtained through the use of the software R Studio version 3.6.1 (www.R-project.org). All negative and positive controls were performed on multiple replicates at least 3 times in each cell line. All experiments were performed at least in duplicates and statistical evaluation was performed using Prism 9 software (https://www.graphpad.com/scientific-software/prism/) or R/Bioconductor. Where applicable, evaluation was done using two-way ANOVA with correction for multiple comparison tests or the Wilcoxon test. *p*-values of <0.05 were considered statistically significant and presented as a value or as **p < 0.01, ***p < 0.001, ****p < 0.0001.

### Reporting summary

Further information on research design is available in the Nature Research Reporting Summary linked to this article.

## Results

### The literature defines various genes predicting PARPi response

**Fig. 1:**
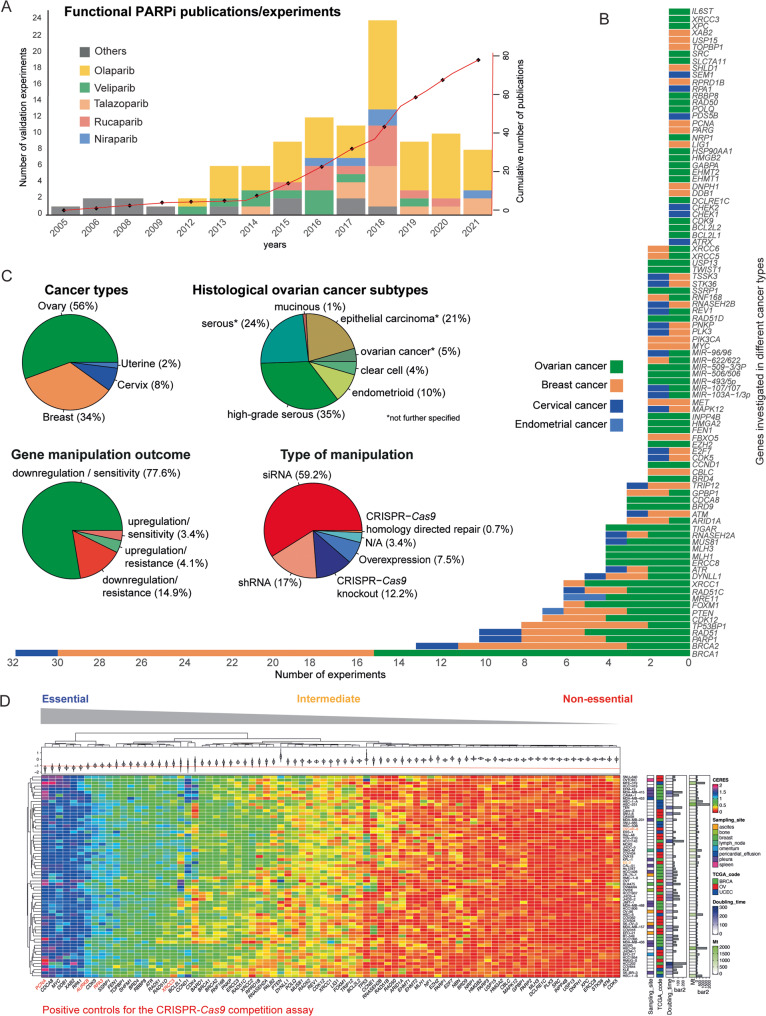
The literature defines various genes associated with altered PARPi response. **A** Cumulative number of publications from 2005 to July 2021 and number of validation experiments using different PARPi. The term orders include 6(5H)-phenanthridinone (PHEN), 4-amino-1,8-naphthalimide (4-ANI), AZD2461, NU1025, AG14361, KU0058948, KU0058684 PARPi’s. **B** Bar and **C** pie charts with number and percentages of validation experiments, type of gene manipulation, and outcome for genes reported to be associated with PARPi response in the different cancer types. **D** Unsupervised clustering and heatmap of gene dependencies based on the CERES score. Each column represents an individual gene selected by the literature search, genes associated with homologous recombination, and frequently altered genes in EOC such as *TP53*, *CCNE1*, and *CCND1* [82]. *PCNA*, *RPA3*, *AURKB*, and *ERCC3* genes previously reported to be essential for cancer cell fitness were used as positive controls [38, 39]. A threshold of <−1 defines the essentiality of the genes.

Fostered by two marker papers in 2005, reporting on remarkable cytotoxicity of PARPi towards cells lacking BRCA functionality [6, 7], several studies suggested potential mechanisms and biomarkers that predict PARPi response. However, most of those were descriptive studies without functionally validating candidate genes. The heterogeneity of the studies regarding candidate genes, models used, and methodological approaches applied, make the overview of the current literature in this field very complex. Thus, we performed a literature search to summarize all studies published between 2005 and July 2021. Considering our inclusion criteria, a total of 79 studies that functionally investigated genes conferring altered PARPi response in gynecological cancers have been published (Fig. 1A and Supplementary Table 3). The majority examined well-described and clinically applied PARPi (olaparib, niraparib, rucaparib, veliparib, and talazoparib) (Fig. 1A). Some studies applied more than one PARPi, most of the time in downstream validation experiments. Our literature search also revealed a total of 93 genes suggested to impact PARPi response. Most of the genes were investigated only once, *with BRCA1, BRCA2, and RAD51* reported in more than 5 publications (Supplementary Table 3, and Supplementary Fig. 4A). More than half of the studies examined PARPi response in EOC models followed by breast cancer while cervical and endometrial cancer were less investigated (Fig. 1C). In regards to EOC, the serous subtype was addressed by the majority of the studies (Fig. 1C). Functional involvement of candidate genes was mostly studied by gene silencing being dominated by experiments using siRNA (59.2%). Importantly, only 7.5% incorporated gene overexpression in the context of PARPi response (Fig. 1C). We further classified the outcome of investigated genes into four different categories; either (1) downregulation or (2) upregulation leads to PARPi sensitivity (80%) and (3) upregulation or, (4) downregulation confers PARPi resistance (20%) (Fig. 1C, Supplementary Fig. 4B).

The discovery and characterization of druggable cancer dependencies are key goals in preclinical research. Although our literature analysis identified 93 genes described in the context of PARPi response (Fig. 1B), their impact on cancer cell fitness defined by altered cell proliferation or survival was less investigated. This bidirectional assessment of gene dependencies is critical to clarify the specific role of the identified genes and exclude a possible general effect on cell survival or proliferation upon gene editing. Thus, we accessed publicly available data derived from CRISPR library screens in ovarian, breast, and endometrial cancer cell lines using the CERES score as a computational readout of gene dependency for cell survival [38]. Interestingly, we observed a large variation of CERES scores for genes identified by our literature search (e.g. *ATR, BARD1, BRCA1, BRCA2, PARP1, RAD51, RAD51B, RAD51D*, and *XRCC3*) (Fig. 1D). Of note, cancer type, doubling time, and mutational burden were not associated with gene-dependent cell fitness (Fig. 1D).

### Establish a functional CRISPR-*Cas9* competition assay enabling assessment of gene dependencies for cancer cell fitness and PARPi response

**Fig. 2:**
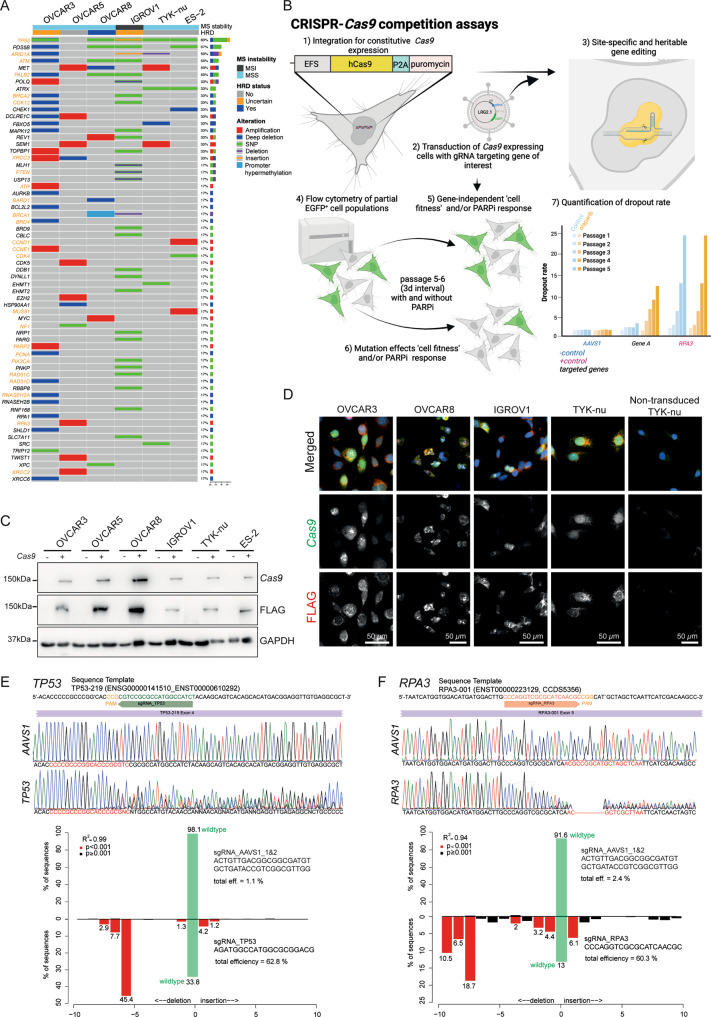
Accessing *Cas9* activity in EOC cell lines through tracking of indels by decomposition (TIDE) assay. **A** Oncoprint for the literature-derived, homologous recombination-associated, and commonly mutated genes, in six EOC cell lines (Data obtained from cBioPortal [83, 84] (only genes harboring an alteration in at least 1 cell line are visualized). Row annotations include homologous recombination and microsatellite stability status of each cell line. Orange highlighted genes were further tested in a CRISPR-*Cas9* cell competition assay. **B** Depiction of the competition assay including assessment and quantification of alterations on cell fitness and PARPi response conferred by gene editing. Dropout values represent the fold-change of EGFP^+^ cells for up to six continuous cell culture passages evaluated by flow cytometry every 3–4 days relative to the EGFP^+^ percentage at passage 0. Passage 0 was measured 3 days after lentiviral transduction with gRNA encoding plasmids. Guide RNAs targeting the human genome safe harbor *AAVS1* (non-essential) and *RPA3* or *PCNA* genes (essential) were used as negative and positive controls throughout all experiments performed, respectively. **C**, **D** Representative Western blot and immunofluorescence images of puromycin selected *Cas9* expressing EOC cell lines. FLAG was evaluated to confirm the intact integration of the *Cas9*-puromycin cassette. **E**, **F** Representative Sanger DNA sequencing for assessment of site-specific mutagenesis using gRNAs targeting **E** *TP53* and **F** *RPA3* exemplified in OVCAR8-*Cas9*^+^. Highlighted regions in red indicate +/−10 nucleotides surrounding the *Cas9* cutting site. Corresponding bar chart with the percentage of indels evaluated by TIDE analysis with an overall efficiency of 62.8% and 60.3% for *TP53* and *RPA3*, respectively. Illustrations were created with BioRender.

In order to investigate the role of the most promising candidate genes in PARPi sensitivity together with cancer cell fitness, we selected six well-annotated EOC cell lines covering a broad spectrum of genetic aberrations observed in EOC patients (Fig. 2A), including *BRCA* mutated and non-mutated cell lines as well HR proficient and deficient cells, and established a CRISPR-*Cas9* cell competition assay. Here, *Cas9*-guide RNA-induced insertions and deletions (indels) in EOC cells may affect cell fitness in a gene-dependent manner and consequently outcompete with non-gRNA transduced cells during continuous cell passaging for up to 24 days (Fig. 2B). Cells were lentivirally transduced and selected for constitutive *Cas9* protein expression (Fig. 2C, D). Next, *Cas9*^*+*^ cells were further transduced with LRG2.1 plasmid encoding guide RNAs (gRNAs) targeting the gene of interest (Supplementary Table 1) and fluorescent proteins, either enhanced green fluorescent protein (EGFP) or mOrange. To assess the efficacy of the CRISPR system, we transduced *Cas9* + cells with gRNAs independently targeting *TP53* and *RPA3;* both previously identified as non-essential and essential genes, respectively [38, 39]. Guide RNAs targeting the human genome safe harbor *AAVS1* were used as negative controls in all assays performed. Next, we analyzed the target *loci* by Sanger DNA sequencing. Tracking of indels by decomposition (TIDE) analysis, which decomposes raw sequencing traces into linear combinations of indel mutations [37] revealed quantitative indels with a heterogenous efficiency and correlated with the transduction efficiency of the cell lines tested (Fig. 2E, F and Supplementary Fig. 5A, B). Of note, we observed a reduction over time in the percentage of cells harboring indels in the *RPA3* target *locus* co-occurring with a decrease in the percentage of fluorescent/ RPA3_gRNA positive cells (Supplementary Fig. 5B). To further verify this approach, we designed gRNAs against additional pan-essential genes *AURKB*, *ERCC3*, and *PCNA* [38, 39]. As expected, guides targeting those genes showed an increased dropout rate of up to 120-fold indicating an overall impact on cell fitness. In contrast, gRNAs targeting *TP53* and *AAVS1* exhibited a marginal dropout rate over six passages in culture (Supplementary Fig. 1A–E). Considering the variations in the dropout rate in the non-essential genes among different *Cas9*^+^ cell lines we established a threshold of 1.7 dropout rate to define the essentiality of the tested genes. Importantly, possible differences in the dropout rate observed for the different genes/guide RNAs were not confounded by the initial percentage of fluorescent positive cells (Supplementary Fig. 1F).

### *Cas9*-mediated gene deletion of *BRCA1* and *BRCA2* increases sensitivity to olaparib in a cell line-dependent manner

**Fig. 3:**
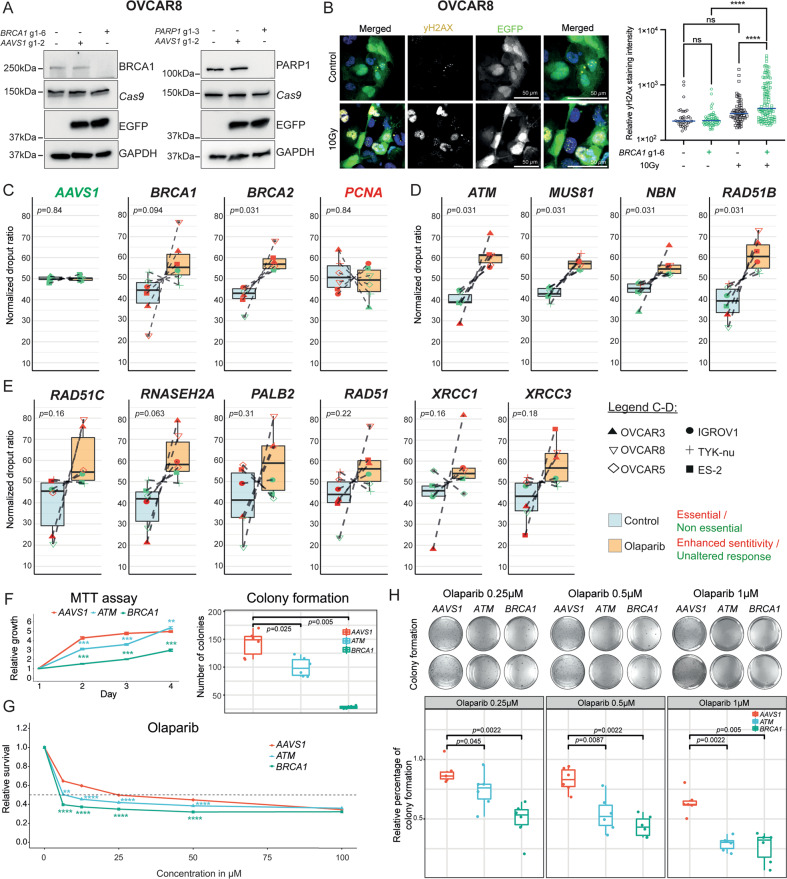
Cancer cell fitness and acquired olaparib sensitivity upon genetic deletion of specific genes. **A** Representative Western blot confirming the loss of BRCA1 and PARP1 expression upon site-specific deletion in OVCAR8-*Cas9*^+^. **B** Gene-editing of *BRCA1* impairs the DNA repair function. DNA damage was assessed by yHA2X staining in EGFP^+^ (sgRNA specific to *BRCA1*) and EGFP^-^ cells (non-transduced cells). **C** Boxplots summarizing the impact on olaparib response in cell lines (*n* = 6) upon gene deletion for negative (*AAVS1*), positive (*PCNA*) controls, and clinically relevant genes *BRCA1* and *BRCA2*. The normalized dropout ratio was calculated by dividing the dropout value of the control part by the olaparib-treated part in passage 6. The threshold to define essential genes was delineated as >1.7 dropout rate in the non-treated conditions and is represented in red. Normalized dropout >55%, represents enhanced olaparib sensitivity and is represented in red. Olaparib concentration used was dependent on the cell line with the range of 0.25–1 µM. **D**, **E** Boxplots for targeted genes in all cell lines showing significant (**D**) and cell line-dependent (**E**) dropout upon olaparib treatment. **F** Results derived from MTT assay and matching colony formation assay (*n* = 2 independent experiments), performed in sextuplicate and triplicates, respectively confirming a significant impact of *BRCA1* deletion on cell fitness in OVCAR8-*Cas9*^+^ cells. **G** Dose-dependent survival upon deletion of *AAVS1*, *ATM*, and *BRCA1* in olaparib treated OVCAR8-*Cas9*^+^ cells. **H** Matching colony formation with two representative wells in OVCAR8-*Cas9*^+^ cells with quantified colonies derived from two independent experiments. *p*-values were calculated by two-way ANOVA followed by Tukey’s multiple comparison test for **B** or Wilcoxon test for **C**–**H**, NS non-significant and **p < 0.01, ***p < 0.001, ****p < 0.0001. See also Supplementary Figs. 6 and 7.

We used immunodetection to verify that the designed sgRNAs targeting genes of interest result in reduced protein expression in OVCAR8-*Cas9*^+^ cells transduced with sgRNAs targeting *BRCA1* and *PARP1*. In addition, we confirmed that *BRCA1*-edited cells had an impaired DNA repair capacity, demonstrated by persistent DNA damage (elevated yH2AX staining) after irradiation as compared with the controls (Fig. 3A, B). To assess whether the CRISPR-*Cas9* cell competition assay can be used to predict PARPi response upon genetic deletion, we initially examined the effect of gRNAs (*n* = 6 per gene) targeting *BRCA1* and *BRCA2*, the most frequently single gene alterations used to prescribe PARPi in EOC patients [40]. Here, we focused on the response to the first clinically approved and widely used PARPi-olaparib [41]. Interestingly, *BRCA1* mutations confer olaparib sensitivity in a cell line-dependent manner. Of note, loss of *BRCA1* and *BRCA2* already impacted cell fitness in the absence of olaparib in most of the cell lines investigated, including OVCAR8, OVCAR3, and IGROV1 with reported *BRCA1* promoter hypermethylation, chromosomal alteration in *BRCA2* and mutations in *BRCA1/2*, respectively (Figs. 2A, 3C and Supplementary Fig. 6A–G).

### CRISPR-*Cas9* cell competition assay reveals cell line- and gene-dependent effects on cancer cell fitness and acquired olaparib sensitivity

A comprehensive characterization of potentially druggable cancer dependencies is of extreme importance in preclinical research. Although our literature analysis identified 93 genes associated with PARPi response (Fig. 1B), their impact on cancer cell fitness defined by altered proliferation or survival was not clearly addressed. This bidirectional assessment of gene dependencies is critical to clarify the specific involvement of the candidate genes in drug responses and exclude a possible general effect on cell survival and proliferation upon gene editing. To unambiguously evaluate gene-dependencies for PARPi responses, we applied the CRISPR-*Cas9* cell competition assay together with olaparib treatment in six EOC cell lines. Candidate genes were selected based on the following parameters: literature-derived genes, reported in more than one publication (*n* = 14) (Supplementary Fig. 4A) genes associated with homologous recombination (*n* = 13), and frequently altered genes in EOC (*n* = 4). Mutagenesis of candidates resulted in a heterogenous outcome regarding the effect on cell fitness and olaparib response (Fig. 3C, D, Supplementary Fig. 6). We found a significant increase in olaparib sensitivity indicating cell line independency upon loss of *ATM, MUS8*1, *NBN*, and *RAD51B*. Despite not reaching significant differences the genetic deletion of *RAD51C*, *RNASEH2A*, *PALB2*, *RAD51*, *XRCC1*, and *XRCC3* showed increased olaparib sensitivity in at least 3 cell lines test, (with a normalized dropout above 55%) indicating cell line-dependency. However, the enhanced olaparib sensitivity also correlated with an impact on cell fitness for those genes (Figs. 3E, Supplementary Fig. 6B–G). The remaining tested genes (*53BP1*, *ARID1A*, *ATR*, *BARD1*, *BRD4*, *CCND1*, *CCNE1*, *CDK4*, *CDK12*, *FOXM1*, *MRE11*, *NF1*, *PARP1*, *PARP2*, *PARP3*, *PIK3CA*, *PTEN*, *RAD51D*, *RAD52*, *SHLD2*, and *XRCC2*) failed to alter PARPi response upon genetic deletion. The loss of *PARP1* and *PARP3* only altered olaparib sensitivity in OVCAR3 cells without any alteration observed for the loss of *PARP2*. We used additional cell proliferation and survival assays to confirm the data generated by the CRISPR-*Cas9* competition assay regarding the essential role of *BRCA1* and enhanced sensitivity to olaparib and niraparib upon loss of *BRCA1* and *ATM* (Fig. 3F–H and Supplementary Fig. 7). Of note, analysis of publicly available data from the TCGA cohort regarding ovarian serous carcinomas demonstrated that around 40% of the cases have alterations in genes identified in our study as functionally linked with olaparib sensitivity (Supplementary Fig. 8). In addition, cell line dependencies previously reported for *CDK4* and *CCND1* [38] (Fig. 1D) could be also reproduced by our CRISPR-*Cas9* cell competition assay (Supplementary Fig. 6B–G).

### Gene-editing resulting in acquired PARPi sensitivity corroborates with chemotherapy response

**Fig. 4:**
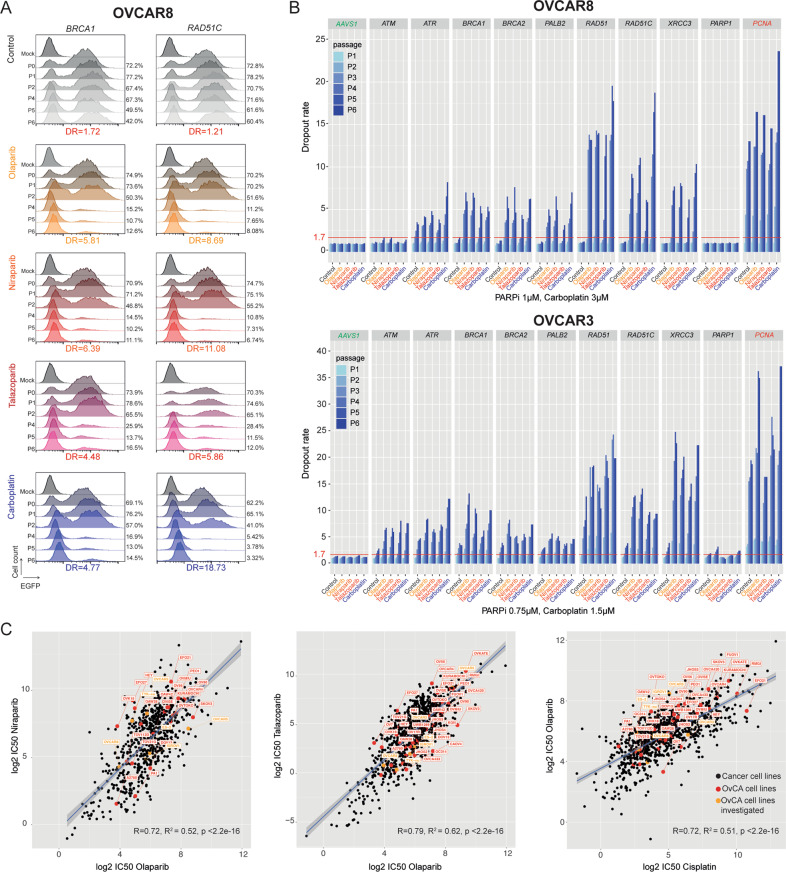
PARPi sensitivity correlates with chemotherapy response. **A** Representative histograms from flow cytometry analysis for the enhanced PARPi sensitivity and carboplatin in cells harboring gRNAs targeting *BRCA1* and *RAD51C*. **B** Histograms for CRISPR-*Cas9* cell competition assay over 6 consecutive passages with increased dropout rates in cells treated with different PARPi and carboplatin. The threshold for essentially was defined as >1.7 dropout rate in the non-treated conditions. **C** Scatter plots demonstrating a positive correlation between olaparib, niraparib, talazoparib, and cisplatin. Data obtained from the DepMap portal (accessed in January 2022) R and R^2^ indicate the correlation coefficient and square correlation coefficient, respectively.

Defects in homologous recombination have been associated not only with PARPi sensitivity but also with DNA-damaging agents (e.g. platinum compounds) [20, 21] and platinum sensitivity in high-grade serous EOC is a strong clinical predictor for the benefit of PARPi treatment [22, 23]. Despite the outstanding benefits of PARPi regarding progression-free survival in high-grade serous EOC patients, it remains undefined which molecular characteristics stratify the best PARPi response. Therefore, to examine the specificity of the enhanced olaparib sensitivity identified upon gene editing, we selected the top nine candidates (*ATM, ATR, BRCA1, BRCA2, PALB2, RAD51, RAD51C, PARP1*, and *XRCC3)* for further validation in combination with niraparib, talazoparib, and carboplatin. The different PARPi were selected based on their clinical application and PARP trapping potency [42]. We observed similar dropout rates for all the tested genes in a cell line- and compound-independent manner (Fig. 4A, B). Surprisingly, the genetic loss of *PARP1* failed to induce resistance in all the compounds tested. In the same line, talazoparib, the most potent PARP1 trapping inhibitor [42] failed to induce an additional increase in the dropout as compared with the other less potent PARPi tested.

To evaluate whether the observed effects were specific for ovarian cancer models or a more generalizable and cancer type-independent effect, we accessed publicly available data regarding drug responses on a broad spectrum of cancer cell lines. As shown in Fig. 4C, the IC_50_ of olaparib was strongly positively correlated with response to niraparib, talazoparib, and cisplatin. Taken together, our functional screening together with publicly available data suggests a general mechanism of response for drugs that depend on DNA repair capacity for their efficacy, demonstrating the difficulty to identify specific predictive biomarkers for PARPi response.

### *CDK12* is an essential gene for cell survival in EOC cell lines

**Fig. 5:**
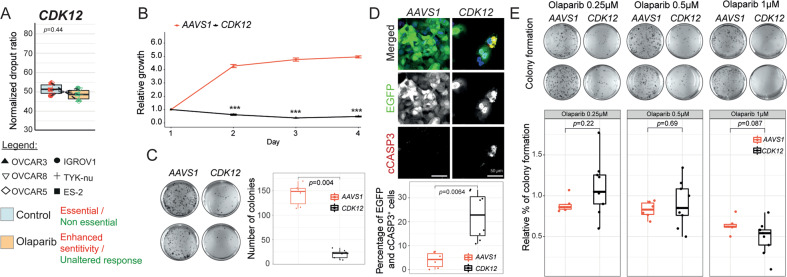
*CDK12* is an essential gene for ovarian cancer cell fitness without altering olaparib sensitivity. **A** Boxplot summarising the unaltered response to olaparib in six EOC cell lines upon *CDK12* editing. **B** Line chart and **C** boxplot showing a significant decrease in cell growth and colony formation capacity in *CDK12*-edited OVCAR8-*Cas9*^+^ cells. **D** Representative immunofluorescence images and the corresponding quantification with a significant increase in the percentage of apoptotic cells (cCASP3+) in *CDK12*-edited OVCAR8-*Cas9*^*+*^ cells. **E** Colony formation with quantified colonies for dose-dependent survival upon deletion of *AAVS1* and *CDK12* in OVCAR8-*Cas9*^+^ derived from at least two independent experiments, *p*-values are calculated by Wilcoxon test, ***p < 0.001.

We found that *CDK12*, a previously reported gene conferring PARPi sensitivity in ovarian [43] and breast cancer [44] models, is an essential gene in all the cell lines tested without altering response to olaparib in our CRISPR-*Cas9* competition assay (Supplementary Fig. 6B–G). To further verify *CDK12* as an essential gene for EOC cell fitness and a potential new target for therapy in ovarian cancer patients, we performed additional MTT, clonogenic, and immunofluorescence assays in cells harboring sgRNAs targeting *CDK12*. As shown in Fig. 5 and Supplementary Fig. 9, we verify that *CDK12* is essential for cell proliferation and cell survival as exemplified by the significant increase in the apoptotic marker cleaved caspase 3 in cells harboring sgRNAs targeting *CDK12* as compared to *AAVS1*. In addition, the loss of *CDK12* resulted in unaltered olaparib sensitivity evaluated by colony formation assay, confirming results obtained from the CRISPR-*Cas9* competition assay (Fig. 5E).

## Discussion

Several clinical trials have shown that PARPi therapy can be beneficial to a wider group of patients beyond *BRCA* mutations and HR-deficiency (HRD) [42]. In the forefront therapy setting, four phase III clinical trials (SOLO-1 [45], PAOLA-1/ENGOT-OV25 [46], PRIMA/ENGOT-OV26 [19] and VELIA/GOG-3005 [47]) demonstrate efficacy as first-line maintenance of PARPi for newly diagnosed EOC. Interestingly, biomarker sub-analysis of PRIMA, PAOLA-1, and VELIA trials revealed PARPi efficacy, even if to a lower extent, in the *BRCA*-wt/HRD positive population. However, only PRIMA and VELIA show an increased benefit in patients without *BRCA* mutation or HRD. In the recurrent setting accompanying patients with relapse after one or more lines of chemotherapy, Study 19 was the first to demonstrate that olaparib maintenance significantly improves progression-free survival in *BRCA*wt platinum-sensitive high-grade EOC [22]. Afterwards, NOVA [18], SOLO2 [48]^,^ and ARIEL 3 [49] trials demonstrated high efficacy for niraparib, olaparib, and rucaparib, respectively, in platinum-sensitive EOC in all three populations analyzed (*BRCA*-mutated, *BRCA*wt/HRD positive, *BRCA*wt/HRD negative) with the greatest benefit for the *BRCA*-mutated tumors. Based on these results, PARPi were also approved as maintenance therapy for platinum-sensitive recurrent EOC, and importantly, irrespective of biomarker status. Thus, clinically defined platinum sensitivity appears as the most reliable predictive factor for enhanced PARPi response. However, in frontline therapy, when platinum sensitivity is unknown, the clinical decision is still driven by *BRCA* and HRD status. MyChoice® CDx and Foundation Medicine’s FoundationFocus® CDx the two clinically approved HRD tests, only reflect a snapshot of the HR status failing to evaluate the dynamic changes that may occur during tumor evolution and treatment [13, 14]. Considering the characteristics of these tests, which are based on the “effect” of HR deficiency rather than on the “cause”, there are ongoing efforts to provide new and validated alternatives to optimize the clinical benefit of PARPi [50]. In regards to the non-*BRCA* HR-related genes, current ESMO guidelines also recommend further research, since there is still not sufficient evidence to predict PARPi response based on panels of individual genes [51].

To shed light on the predictive power of single gene aberrations conferring PARPi sensitivity, we performed a literature review and identified 93 genes reported to be involved in altered PARPi responses in gynecological tumors. However, we found inconsistent results regarding the outcome of gene manipulation and altered PARPi responses for the *RNASEH2A* [52, 53] and *PTEN* [54–57] in our literature review. One possible justification for the inconsistent results is that the studies used different cancer cell lines with distinct BRCA functionality. In fact, our mutagenesis screen identified enhanced olaparib sensitivity upon *PTEN* loss only for OVCAR3 and IGROV1 with already reported alterations in *BRCA1/2* genes. On the other hand, the altered olaparib response upon genetic loss of *RNASEH2A* was not associated with the *BRCA* status of the cell lines in our screen, suggesting a cell line-dependent effect rather than the *BRCA* status.

From the 33 genes assessed by our CRISPR-*Cas9* mutagenesis assay, we identified *ATM*, *MUS81*, *NBN*, *BRCA2*, and *RAD51B* as genes significantly associated with olaparib sensitivity in a cell-independent manner suggesting that mutations in those genes leading to loss of function enhance PARPi response*. ATM* is a master regulator of DNA damage response [58], and altered ATM expression or function has been reported to be associated with PARPi sensitivity in several tumor contexts [59–61]. However, to the best of our knowledge, our study is the first to provide functional evidence that the loss of *ATM* results in increased PARPi sensitivity. Despite additional validation studies being necessary, our data suggest that besides *BRCA1/2* the genetic screen of *ATM*, *MUS81*, *NBN*, and *RAD51B* has the potential to increase the number of EOC patients that could benefit from PARPi therapy. We also identified that *Cas9*-mediated loss of *PALB2*, *RAD51, RAD51C, RNASEH2A, XRCC1, and XRCC3* conferred olaparib sensitivity in at least 3 out of 6 cell lines. These results suggest that to some extent the effect of those genes on olaparib sensitivity can be dependent on several factors intrinsic to the cell lines such as genetic and epigenetic backgrounds [62, 63], and clinical parameters from the original tumors where the cell lines were established (sampling site and pretreatment) [64]. Therefore, additional studies are necessary to fully elucidate those dependencies for increased PARPi sensitivity in each gene.

We found apparent inconsistent results regarding the role of *53BP1*, *ARID1A*, *ATR*, *BARD1*, *BRD4*, *CCND1*, *CCNE1*, CDK*4*, *CDK12*, *FOXM1*, *MRE11*, *NF1*, *PARP1*, *PARP2*, *PARP3*, *PIK3CA*, *PTEN*, *RAD51D*, *RAD52*, *SHLD2*, *XRCC2* on olaparib sensitivity. Although we cannot rule out that the inconsistencies between our results and the literature may be linked to the selection of different cell lines with differential genetic backgrounds, BRCA, and HR status, a possible justification for these inconsistencies may also rely on the novelty of our study by addressing the effect of gene modification in cell fitness together with altered PARPi response. This bidirectional assessment of gene dependencies has been less addressed by previous studies, which we see as critical to clarify the specific role of the identified genes in altering drug responses and exclude a possible general effect of cell survival and proliferation upon gene editing. Moreover, in the CRISPR-*Cas9* competition assay cells are analyzed shortly after gRNA transfection allowing them no time to adapt to the loss of the target gene, which can happen by gene downregulation assays using shRNA or classical gene knockout experiments using CRISPR-*Cas9* which may also face the problem on clonal effects. In addition, we cannot rule out the possibility that the genetic loss of a single gene in an in vivo scenario alters the cell response to PARPi, either by interference with the tumor microenvironment or triggering immune response as pointed out by recent publications [65–68].

Through a series of functional assays, we identified *CDK12* as a conservative vulnerability in EOC cell lines without altering the olaparib response. These results suggest *CDK12* as a potential new target for targeted therapy in EOC being in line with the literature on other cancer types [69, 70]. On the other hand, *CDK12* has been previously reported to be associated with altered PARPi response in ovarian cancer [71], and several studies reported synergistic effects on CDK12 inhibition with PARPi [43, 72, 73]. This apparent discrepancy between our results and the previous studies reinforces the importance of our study in providing a systematic assessment of gene dependencies not only for PARPi responses but also for cell fitness. Notably, most of the studies focused on the use of a class of inhibitors targeting multiple cyclin-dependent kinases (CDK)s suggesting that previously reported results may be linked with the inhibition of multiple CDKs rather than *CDK12* specific.

The application of PARPi was demonstrated to be successful in several tumor types [74, 75], but their efficacy has always been shown in comparison to the placebo, except for the phase III randomised OlympiAD trial (NCT02000622), where olaparib showed superiority in prolonging progression-free survival when compared to chemotherapy in *BRCA* mutated HER2 negative metastatic breast cancer [76]. Moreover, the efficacy of the different PARPi have not been compared, and the outcomes of the clinical trials testing different PARPi are not meaningfully comparable, due to differences in study designs regarding control arms, and different timing of PARPi initiation, and duration of therapy [77]. In addition, preclinical studies show that PARPi differs in PARP trapping potency [78], pharmacokinetics, and pharmacodynamics [79, 80]. Therefore, to examine the specificity of the enhanced olaparib sensitivity identified upon gene-editing, we selected the nine candidates for further validation in combination with niraparib, talazoparib, and carboplatin. Our experimental setup suggests a general mechanism of response supporting the strong positive correlation between platinum sensitivity and increased PARPi benefits seen in multiple clinical trials [18, 48, 49, 81]. Interestingly, through classical MTT assays, we observed an enhanced potency when applied niraparib compared to a less potent PARPi olaparib (Fig. 3G and Supplementary Fig. 7). However, we did not observe an increased sensitivity after gene editing of the different target genes with increased trap potency of the different PARPi, suggesting that the efficacy of the different PARPi is possibly linked with their differential polypharmacology [79] rather than with single gene alteration.

To the best of our knowledge, this is the first study functionally testing the role of more than 30 genes regarding cancer cell fitness together with PARPi response. This study used a CRISPR-*Cas9* competition assay, testing gene-dependencies for PARPi response, and cell fitness excluding possible general effects on cell survival and proliferation upon gene-editing of the candidate genes. The non-consideration of the potential effects on cell fitness induced by gene editing can lead to a misinterpretation of the results and reduce the efficacy of promising “in vitro” validated candidates when transferred at a clinical level [19].

## Supplementary information

Original Data File

Reporting Summary

Supplementary figure 1

Supplementary figure 2

Supplementary figure 3

Supplementary figure 4

Supplementary figure 5

Supplementary figure 6

Supplementary figure 7

Supplementary figure 8

Supplementary figure 9

Supplementary figure legends

Supplementary table 1

Supplementary table 2

Supplementary table 3

## Data Availability

The data generated in this study are available within the article and its supplementary data files.

## Code availability

Codes are available from the corresponding author upon reasonable request.
