# Supplementary figures and images for: Overlapping gene dependencies for PARP inhibitors and carboplatin response identified by functional CRISPR-Cas9 screening in ovarian cancer
Source: Cell Death Dis. 2022 Oct 28;13(10):909. doi: 10.1038/s41419-022-05347-x (PMC9616819; doi:10.1038/s41419-022-05347-x)

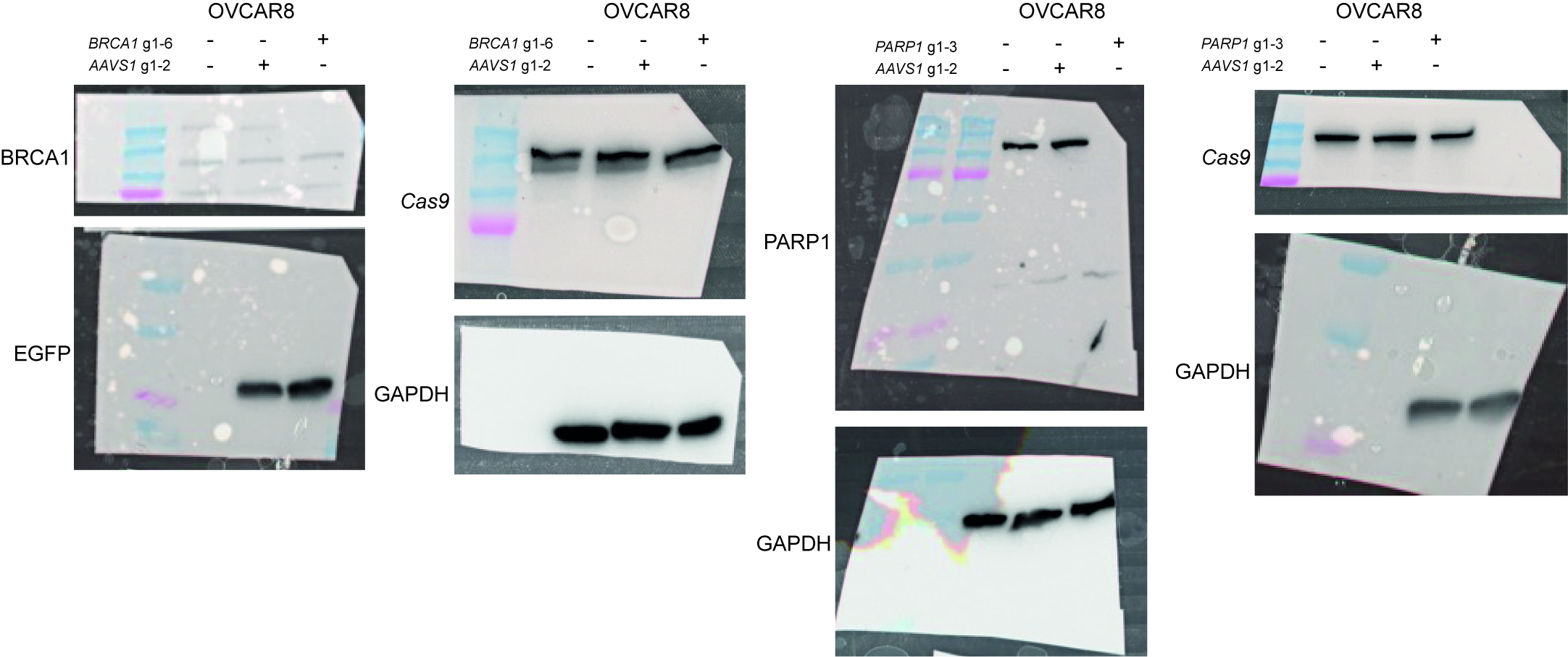

Supplement: Supplementary file 1 — Original Data File [file 41419_2022_5347_MOESM1_ESM.tif]

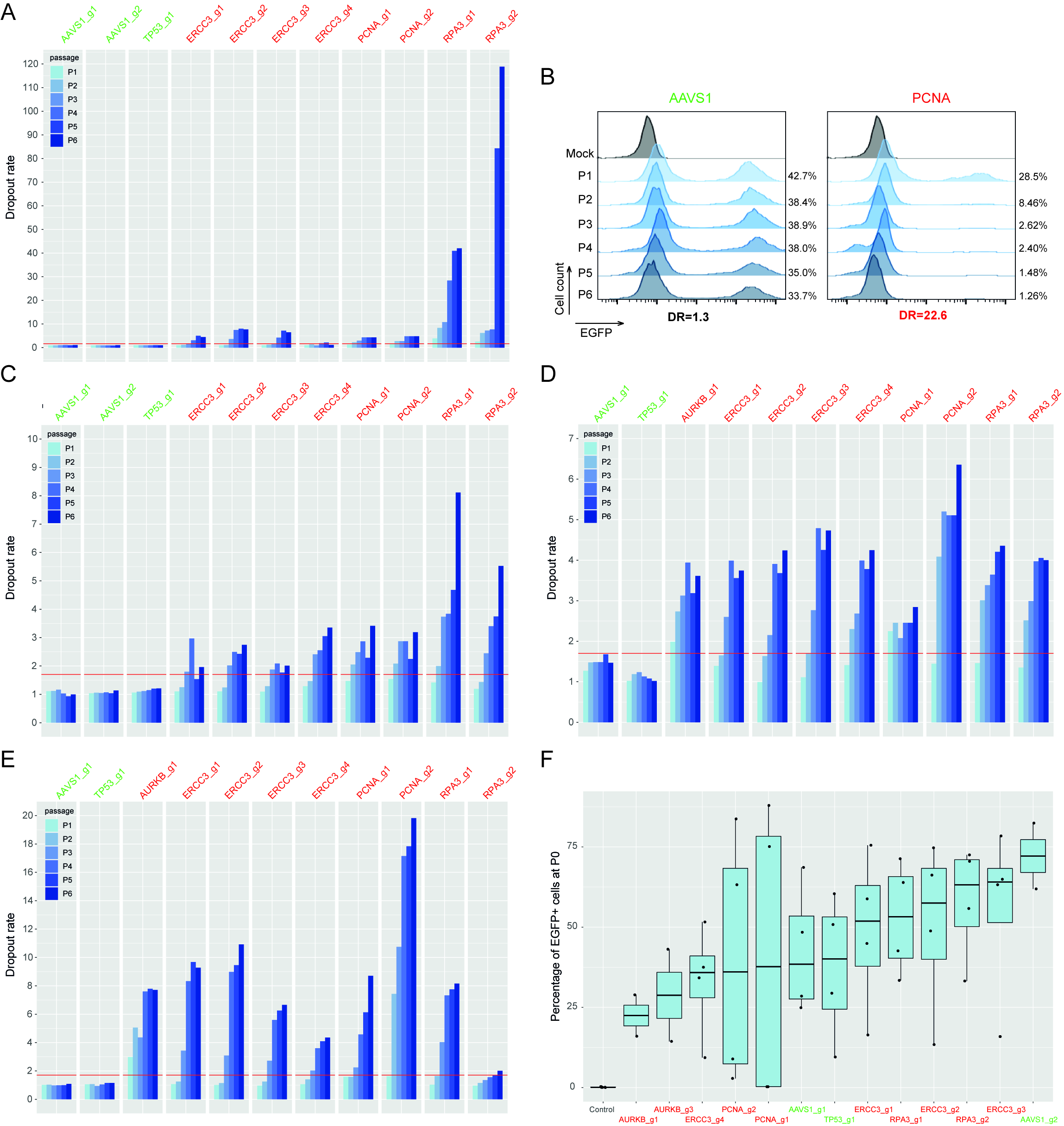

Supplement: Supplementary file 3 — Supplementary figure 1 [file 41419_2022_5347_MOESM3_ESM.tif]

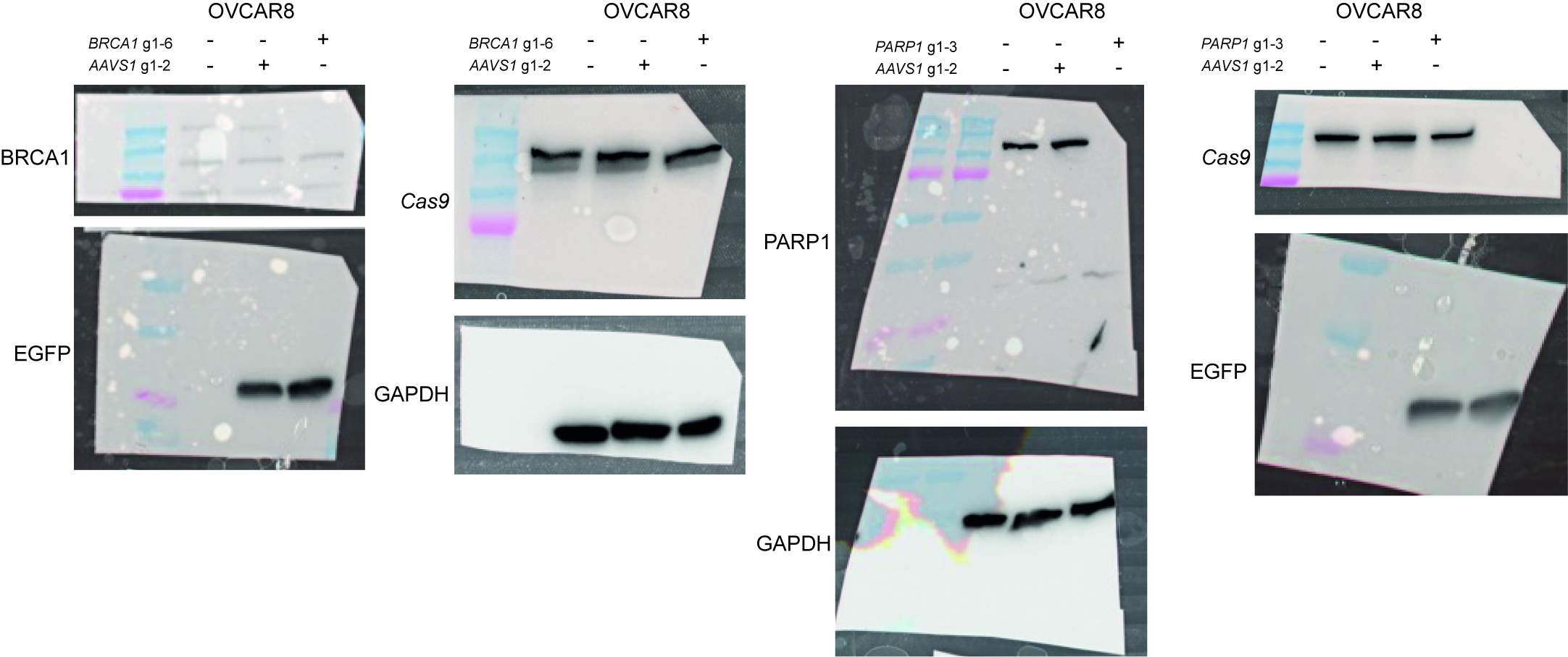

Supplement: Supplementary file 4 — Supplementary figure 2 [file 41419_2022_5347_MOESM4_ESM.tif]

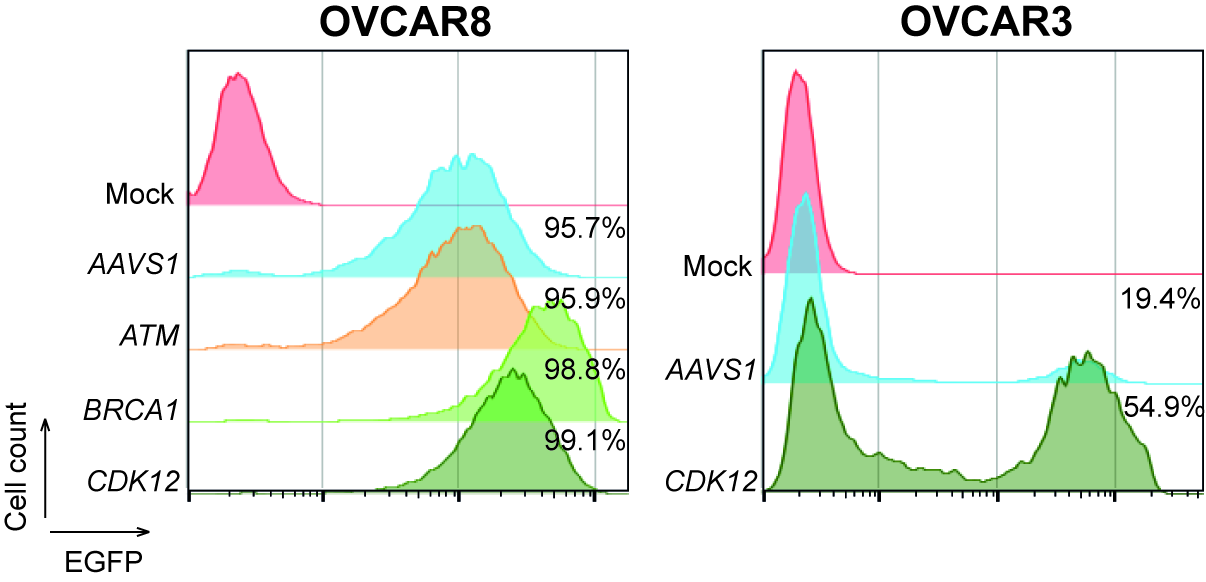

Supplement: Supplementary file 5 — Supplementary figure 3 [file 41419_2022_5347_MOESM5_ESM.tif]

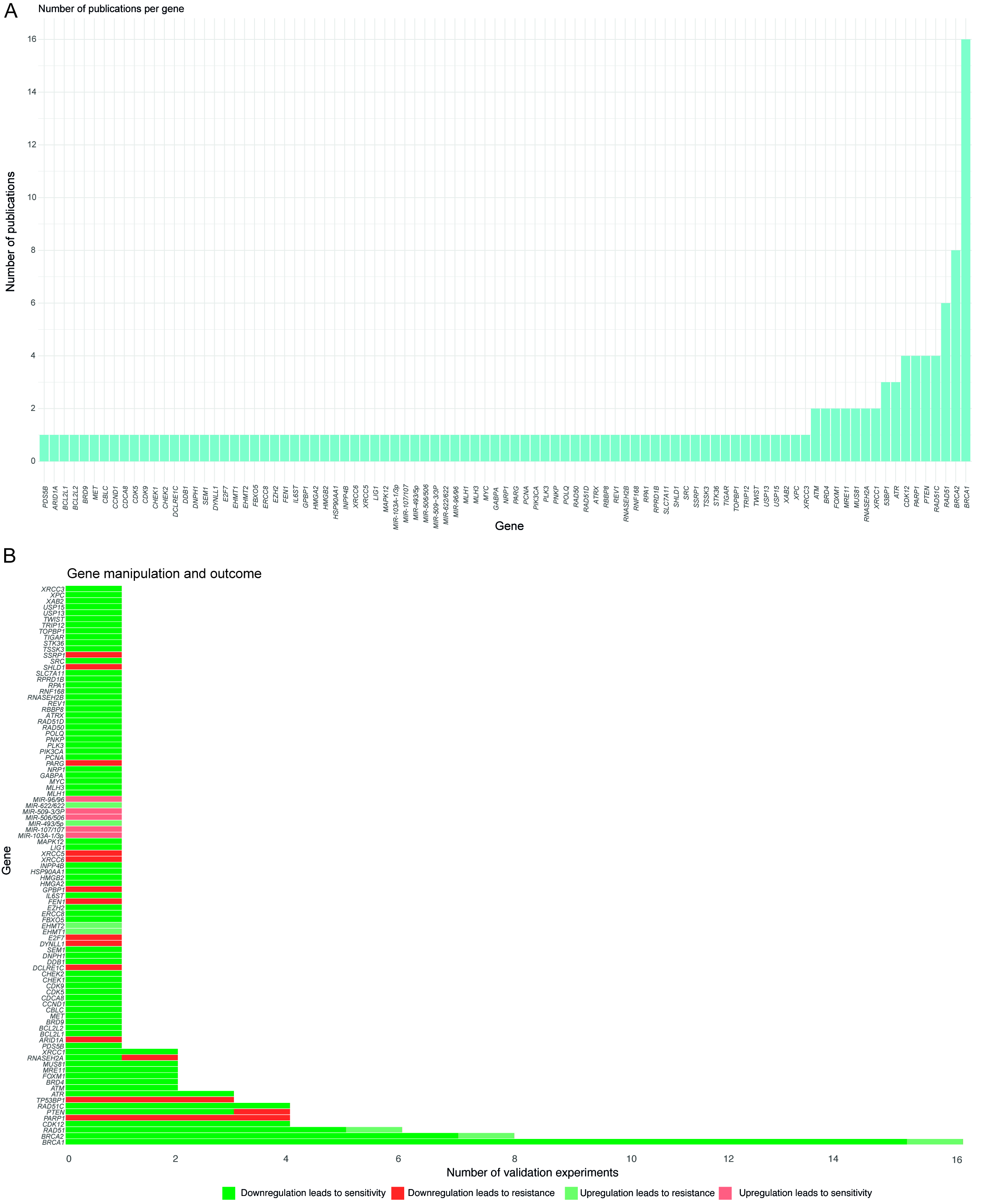

Supplement: Supplementary file 6 — Supplementary figure 4 [file 41419_2022_5347_MOESM6_ESM.tif]

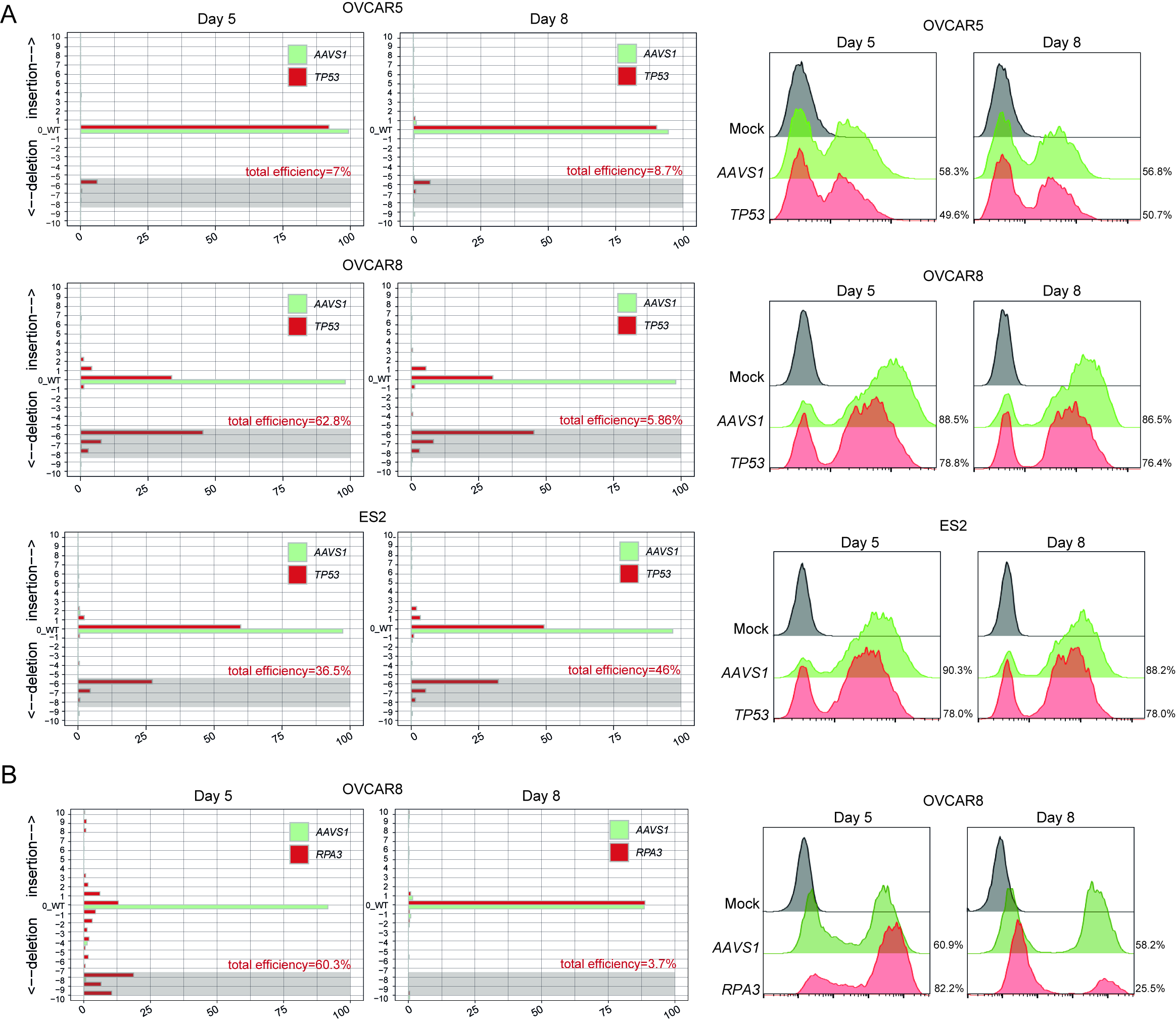

Supplement: Supplementary file 7 — Supplementary figure 5 [file 41419_2022_5347_MOESM7_ESM.tif]

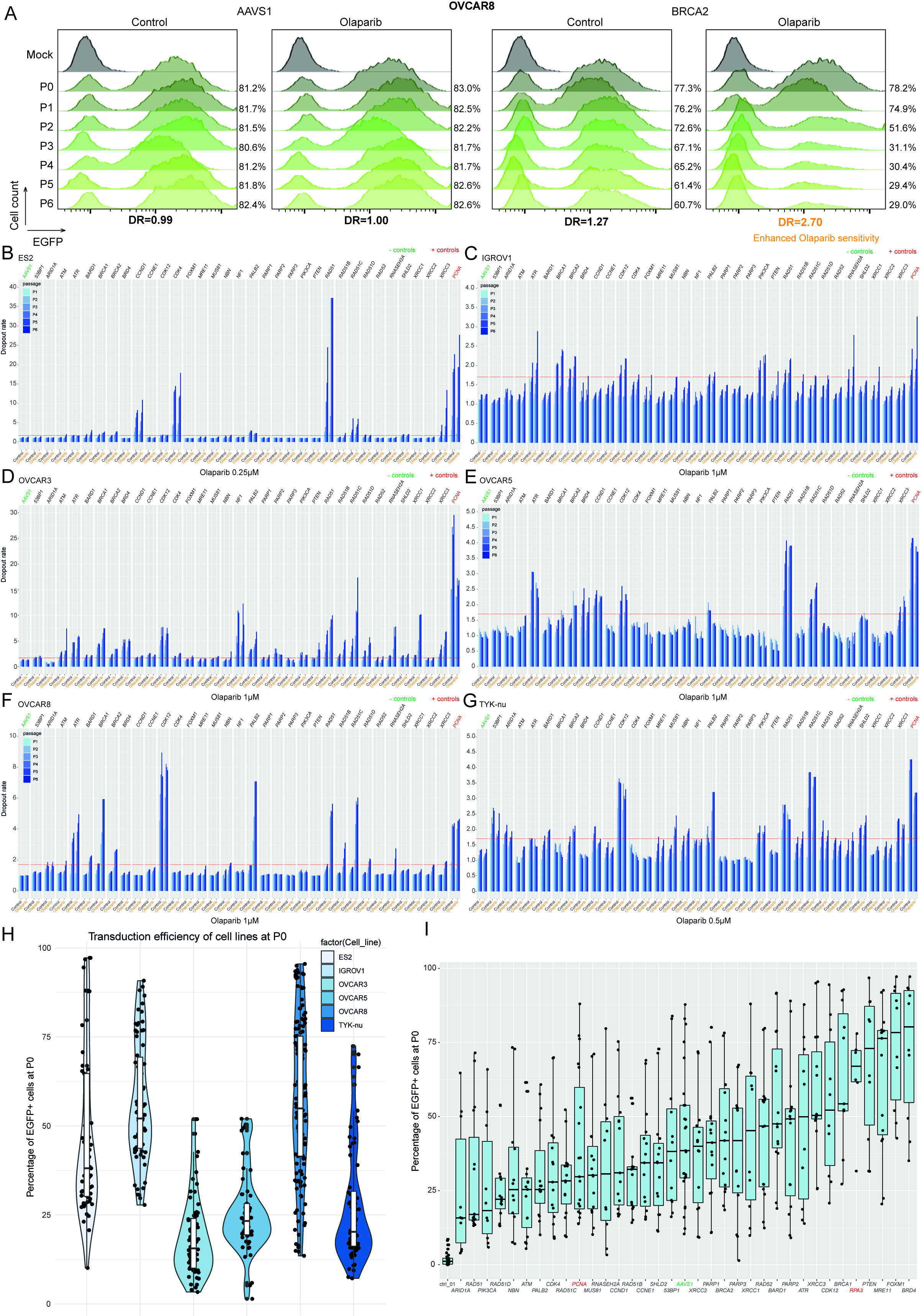

Supplement: Supplementary file 8 — Supplementary figure 6 [file 41419_2022_5347_MOESM8_ESM.tif]

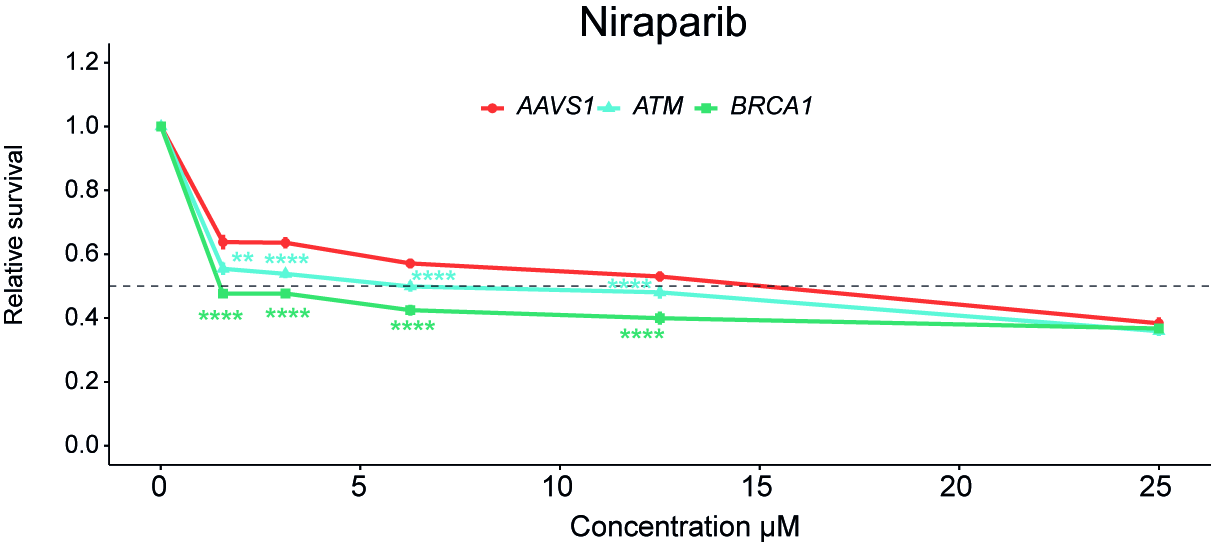

Supplement: Supplementary file 9 — Supplementary figure 7 [file 41419_2022_5347_MOESM9_ESM.tif]

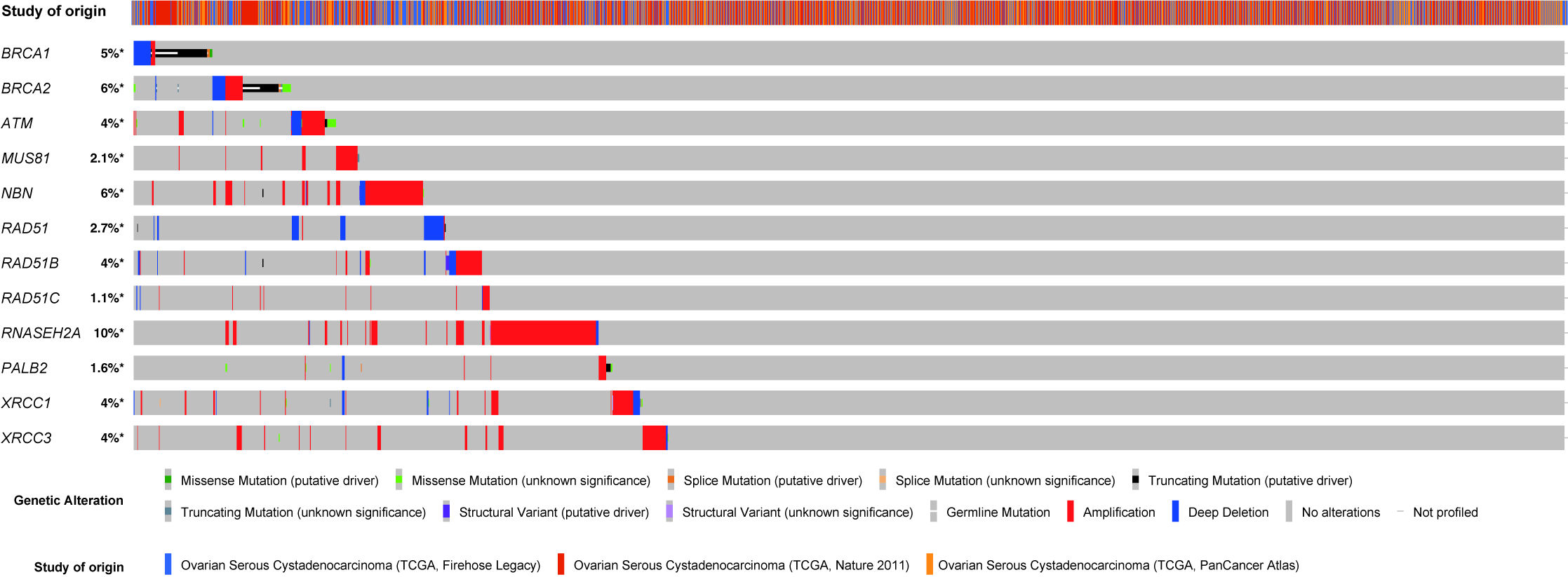

Supplement: Supplementary file 10 — Supplementary figure 8 [file 41419_2022_5347_MOESM10_ESM.tif]

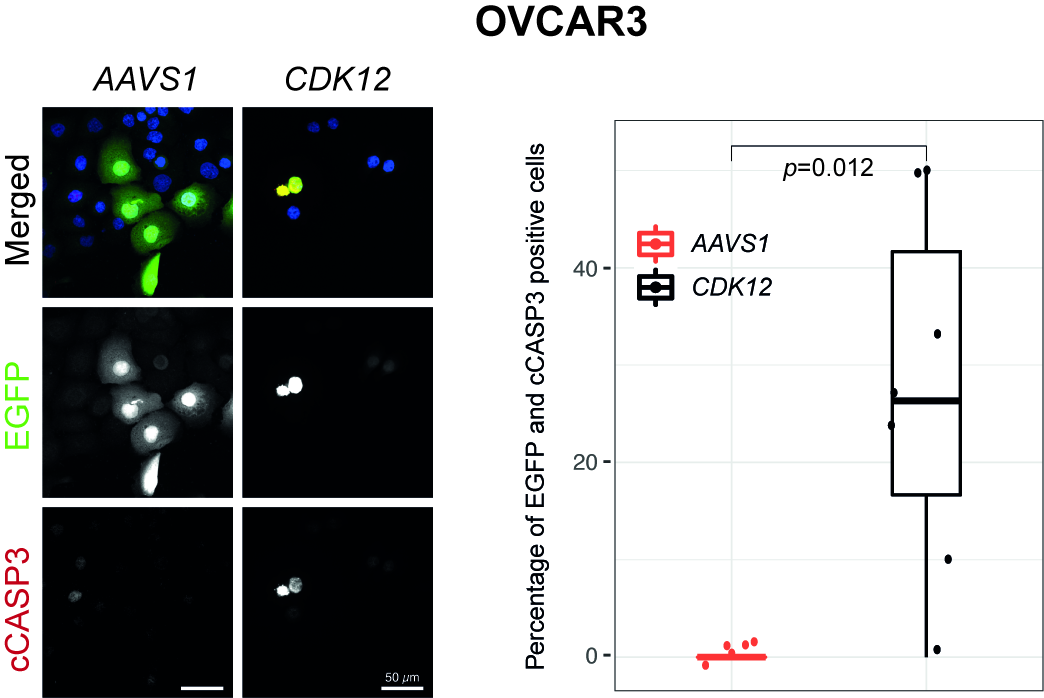

Supplement: Supplementary file 11 — Supplementary figure 9 [file 41419_2022_5347_MOESM11_ESM.tif]
