# Supplementary material for: Overlapping gene dependencies for PARP inhibitors and carboplatin response identified by functional CRISPR-Cas9 screening in ovarian cancer
Source: Cell Death Dis. 2022 Oct 28;13(10):909. doi: 10.1038/s41419-022-05347-x (PMC9616819; doi:10.1038/s41419-022-05347-x)
Supplement: Supplementary file 12 — Supplementary figure legends [file 41419_2022_5347_MOESM12_ESM.docx]

Figure legends:

**Supplementary figure 1. CRISPR-*Cas9* screens for cell line independent effects on cell fitness for essential and non-essential genes**. **A-E)** Barchart for CRISPR-*Cas9* cell competition assay over 6 consecutive passages for non-essential (green) and essential genes (red). The threshold for essentially was defined as >1.7 dropout rate. **F)** Boxplots for the cell-independent transduction efficiency of guide RNAs targeting individual genes of interest.

**Supplementary figure 2. Original images from the Western Blots experiments.**

**Supplementary figure 3.** **Transduction efficiency of OVCAR8-*Cas9*^+^ and OVCAR3-*Cas9^+^* cells for targeted genes**. R*e*presentative histograms derived from flow cytometry for OVCAR8-*Cas9*^+^ and OVCAR3-*Cas9*^+^ cells showing the percentage of cells harbouring gRNAs targeting *AAVS1* (n=2), *ATM* (n=3), *BRCA1* (n=6), and *CDK12* (n=3), EGFP^+^ cells.

**Supplementary figure 4. The literature review identifies 93 genes associated with altered PARPi response.** **A** and **B**) Bar plots for A) the number of publications and B) the outcome of the gene manipulation regarding genes identified as functionally linked with altered PARPi responses.

**Supplementary figure 5*.*** **TIDE assay identifies specific *Cas9*-gRNA-induced indels with heterogeneous efficiency.** **A** and **B**) Bar plot with the percentage of indel formation evaluated by TIDE analysis for A) *TP53* and B) *RPA3* after 5 and 8 days of lentivirus transduction. Corresponding histograms from flow cytometry analysis for percentage of EGFP^+^ cells in cell lines harbouring gRNAs targeting *TP53*, *RPA3*, and *AAVS1*.

**Supplementary figure 6. CRISPR-*Cas9* screen identifies cell line-dependent effects on cell fitness and olaparib sensitivity independent of transduction efficiency. A)** Representative histograms from flow cytometry analysis for OVCAR8-*Cas9* cells harbouring gRNAs targeting *AAVS1 (n=2)* and *BRCA2* (n=6) in combination with olaparib treatment (1 µM) and corresponding dropout rates (DR). **B-G)** Histograms for CRISPR-*Cas9* competition assay over 6 consecutive passages with and without olaparib treatment in six *Cas9*^+^ EOC cell lines. The threshold to define essential genes was delineated as  >1.7 dropout rate in the non-treated conditions. **H)** Violin plots of the transfection efficiency of the pooled guide RNAs (n=2 to 6) targeting genes of interest in all cell lines. **I)** Boxplots for the cell-independent transduction efficiency of guide RNAs targeting individual genes of interest.

**Supplementary figure 7. Increased niraparib sensitivity upon loss of *ATM* and *BRCA1*.** Results derived from the MTT assay (n=2 independent experiments, performed in sextuplicate) confirmed a significant impact of *ATM* and *BRCA1* deletion in niraparib sensitivity in OVCAR8-*Cas9*^+^. p-values were calculated by Wilcoxon test, ** p<0.01, ****p<0.0001.

**Supplementary figure 8.  Genes identified by our CRISPR-*Cas9* screen as functionally linked with olaparib sensitivity are frequently altered serous ovarian carcinomas.**

**Supplementary figure 9. *CDK12* is an essential gene for cell survival in OVCAR3.** Representative immunofluorescence images and corresponding quantification show a significant increase in the percentage of apoptotic cells (cCASP3^+^) on *CDK12*-edited OVCAR3-*Cas9^+^* cells derived from 2 independent experiments.
